# Atopic Eczema in Adulthood and Risk of Depression and Anxiety: A Population-Based Cohort Study

**DOI:** 10.1016/j.jaip.2019.08.030

**Published:** 2020-01

**Authors:** Yochai Schonmann, Kathryn E. Mansfield, Joseph F. Hayes, Katrina Abuabara, Amanda Roberts, Liam Smeeth, Sinéad M. Langan

**Affiliations:** a Department of Non-Communicable Disease Epidemiology, Faculty of Epidemiology and Population Health, London School of Hygiene and Tropical Medicine, London, United Kingdom; b Clalit Health Services, Department of Family Medicine, Rabin Medical Center, Petah Tikva, Israel; c Department of Family Medicine, Sackler Faculty of Medicine, Tel-Aviv University, Tel-Aviv, Israel; d Division of Psychiatry, University College London, London, United Kingdom; e Camden and Islington National Health Service (NHS) Foundation Trust, London, United Kingdom; f Department of Dermatology, University of California San Francisco, San Francisco, Calif; g Nottingham Support Group for Carers of Children with Eczema, Nottingham, United Kingdom; h St John's Institute of Dermatology, Guy's & St Thomas' Hospital National Health Service (NHS) Foundation Trust and King's College London, London, United Kingdom; i Health Data Research UK, London, United Kingdom

**Keywords:** Atopic eczema, Atopic dermatitis, Anxiety, Depression, Population-based, Severity, BMI, Body mass index, CPRD, Clinical Practice Research Datalink, HES, Hospital Episode Statistics, HR, Hazard ratio

## Abstract

**Background:**

Atopic eczema is a common and debilitating condition associated with depression and anxiety, but the nature of this association remains unclear.

**Objective:**

To explore the temporal relationship between atopic eczema and new depression/anxiety.

**Methods:**

This matched cohort study used routinely collected data from the UK Clinical Practice Research Datalink, linked to hospital admissions data. We identified adults with atopic eczema (1998-2016) using a validated algorithm, and up to 5 individuals without atopic eczema matched on date of diagnosis, age, sex, and general practice. We estimated the hazard ratio (HR) for new depression/anxiety using stratified Cox regression to account for age, sex, calendar period, Index of Multiple Deprivation, glucocorticoid treatment, obesity, smoking, and harmful alcohol use.

**Results:**

We identified 526,808 adults with atopic eczema who were matched to 2,569,030 without. Atopic eczema was associated with increased incidence of new depression (HR, 1.14; 99% CI, 1.12-1.16) and anxiety (HR, 1.17; 99% CI, 1.14-1.19). We observed a stronger effect of atopic eczema on depression with increasing atopic eczema severity (HR [99% CI] compared with no atopic eczema: mild, 1.10 [1.08-1.13]; moderate, 1.19 [1.15-1.23]; and severe, 1.26 [1.17-1.37]). A dose-response association, however, was less apparent for new anxiety diagnosis (HR [99% CI] compared with no atopic eczema: mild, 1.14 [1.11-1.18]; moderate, 1.21 [1.17-1.26]; and severe, 1.15; [1.05-1.25]).

**Conclusions:**

Adults with atopic eczema are more likely to develop new depression and anxiety. For depression, we observed a dose-response relationship with atopic eczema severity.

***What is already known about this topic?*** Atopic eczema is a common debilitating skin condition. An association between atopic eczema and common mental disorders is well documented, but its nature and temporal direction remain unclear.

***What does this article add to our knowledge?*** Individuals affected with atopic eczema are more likely to develop new depression (14% increased incidence) and anxiety (17% increased incidence). The observed dose-response relationship between atopic eczema severity and depression supports a causal mechanism for the association.

***How does this study impact current management guidelines?*** Recent atopic eczema guidelines comment briefly on the influence of psychological and emotional factors on the clinical course of atopic eczema. Our findings suggest that depression and anxiety should be addressed explicitly in updated guidelines.

## Introduction

Atopic eczema (eczema, atopic dermatitis) is a chronic relapsing inflammatory skin disease. It can cause intense itching and discomfort. Itch and disfiguring lesions result in sleeplessness and social embarrassment, impairing the quality of life of both sufferers and their families.^1^, ^2^ Atopic eczema is common (20% of children and up to 10% of adults in developed countries) and is a major cause of years lost because of disability.^2^, ^3^, ^4^ Emerging evidence suggests that biologic agents, an effective treatment modality for severe atopic eczema,^2^, ^5^, ^6^ may also reduce symptoms of depression and anxiety among people with atopic eczema.^7^

Mental health disorders are one of the leading causes of disability worldwide,^8^ with depression and anxiety together accounting for more than half of that burden.^9^ Depression, manifesting as loss of interest and enjoyment in ordinary things and experiences, affects approximately 4.4% of the global population; anxiety disorders, characterized by excessive fear, anxiousness, or avoidance of perceived threats, affect approximately 3.6%.^10^ Both depression and anxiety are associated with increased morbidity and mortality.^11^, ^12^, ^13^, ^14^, ^15^ Atopic eczema has been shown to be associated with common mental disorders (depression and anxiety) and suicidality in cross-sectional studies that have frequently relied on self-reported exposures and outcomes.^16^, ^17^, ^18^, ^19^, ^20^, ^21^, ^22^, ^23^, ^24^, ^25^ Individuals with atopic eczema may be more likely to experience depression and anxiety through the effects of itch and discomfort, disfigurement, and perceived social-stigmatization^26^, ^27^, ^28^; in addition, poor sleep related to atopic eczema may increase the risk of mental illness.^29^, ^30^ Inflammatory mediators in atopic eczema could also contribute to the development of depression.^22^, ^31^ However, those with depression and anxiety could also be more likely to consult for a physical condition such as atopic eczema. Because longitudinal evidence is scarce and conflicting, the temporality of any association between atopic eczema and depression and anxiety, and whether the relationship changes with increasing atopic eczema severity, remains unclear.^32^, ^33^, ^34^

Insight into the temporal relationship between atopic eczema and depression/anxiety could guide the clinical approach to this vulnerable group with visible and potentially stigmatizing skin disease. Atopic eczema is common, so if people with atopic eczema are indeed at increased risk of new-onset depression or anxiety, then this would suggest: (1) a major population impact; (2) a potential role for targeted mental health screening for individuals with atopic eczema; and (3) the possibility of mental health modification through improved atopic eczema control (eg, using new biologic agents). Therefore, we aimed to investigate the association between atopic eczema and newly diagnosed depression and anxiety, and whether any association increased with increasing atopic eczema severity, through a longitudinal analysis of UK primary care electronic health record data.

## Methods

### Study design and setting

We conducted a cohort study, using routinely collected primary care electronic health record data from practices contributing to the UK Clinical Practice Research Datalink (CPRD) and linked hospital admissions data from the Hospital Episode Statistics (HES) database. The CPRD covers approximately 7% of the UK population, is broadly representative of the general population, and includes demographic information, diagnoses, prescriptions, and secondary care referrals.^35^ Diagnoses are recorded in the CPRD using Read codes,^36^ and have been demonstrated to be valid.^37^, ^38^ The CPRD ensures high-quality data through algorithmic analysis of gaps in data entry and deaths recorded by each practice.^35^ HES includes data on all the National Health Service–funded inpatient hospital stays in England since 1997, including diagnoses recorded using the *International Classification of Diseases, Tenth Revision* coding system.^39^ Linkage to HES data is available in approximately 80% of English CPRD practices. The study period was from January 2,1998, to March 31, 2016.

### Ethical approval

The study protocol was approved by the Independent Scientific Advisory Committee (ISAC) for the CPRD (ISAC protocol no. 16_100RA) and the London School of Hygiene and Tropical Medicine (Reference: 15460). Informed consent was not required, because the study used anonymized data.

### Study population

#### Individuals with atopic eczema and disease severity

Atopic eczema diagnosis was based on a validated algorithm (positive predictive value of 82%) requiring a record of at least 1 diagnostic code for atopic eczema and at least 2 records for atopic eczema therapy.^40^ Systemic glucocorticoids were not included in the validated algorithm to identify atopic eczema, and their use is generally discouraged^41^ (see this article's “Codes and treatments used in algorithm definition of atopic eczema” section in the Online Repository at www.jaci-inpractice.org). Other inclusion criteria were: adults 18 years and older; eligible for HES linkage; registered with a CPRD practice meeting CPRD patient- and practice-level quality control standards; and contribution of valid follow-up time during the study period (January 2, 1998, to March 31, 2016).

To capture the progressive nature of atopic eczema and to avoid immortal-time bias, atopic eczema severity was modeled as a time-updated variable.^42^ We categorized severity into 3, mutually exclusive, progressive categories (mild, moderate, and severe) according to recorded atopic eczema therapy.^5^, ^43^, ^44^ By default, all individuals with atopic eczema were classified as having mild disease. They could be recategorized as having (1) moderate atopic eczema if potent topical steroids or calcineurin inhibitors were prescribed or (2) severe atopic eczema, if there was a record for a referral to a dermatologist, or a record for systemic treatment. Individuals with moderate/severe disease kept their severity category until the end of follow-up and could not be recategorized as having milder disease (see this article's “Codes and treatments used in algorithm definition of atopic eczema” section).

#### Comparison group of individuals without atopic eczema

**Figure E1:**
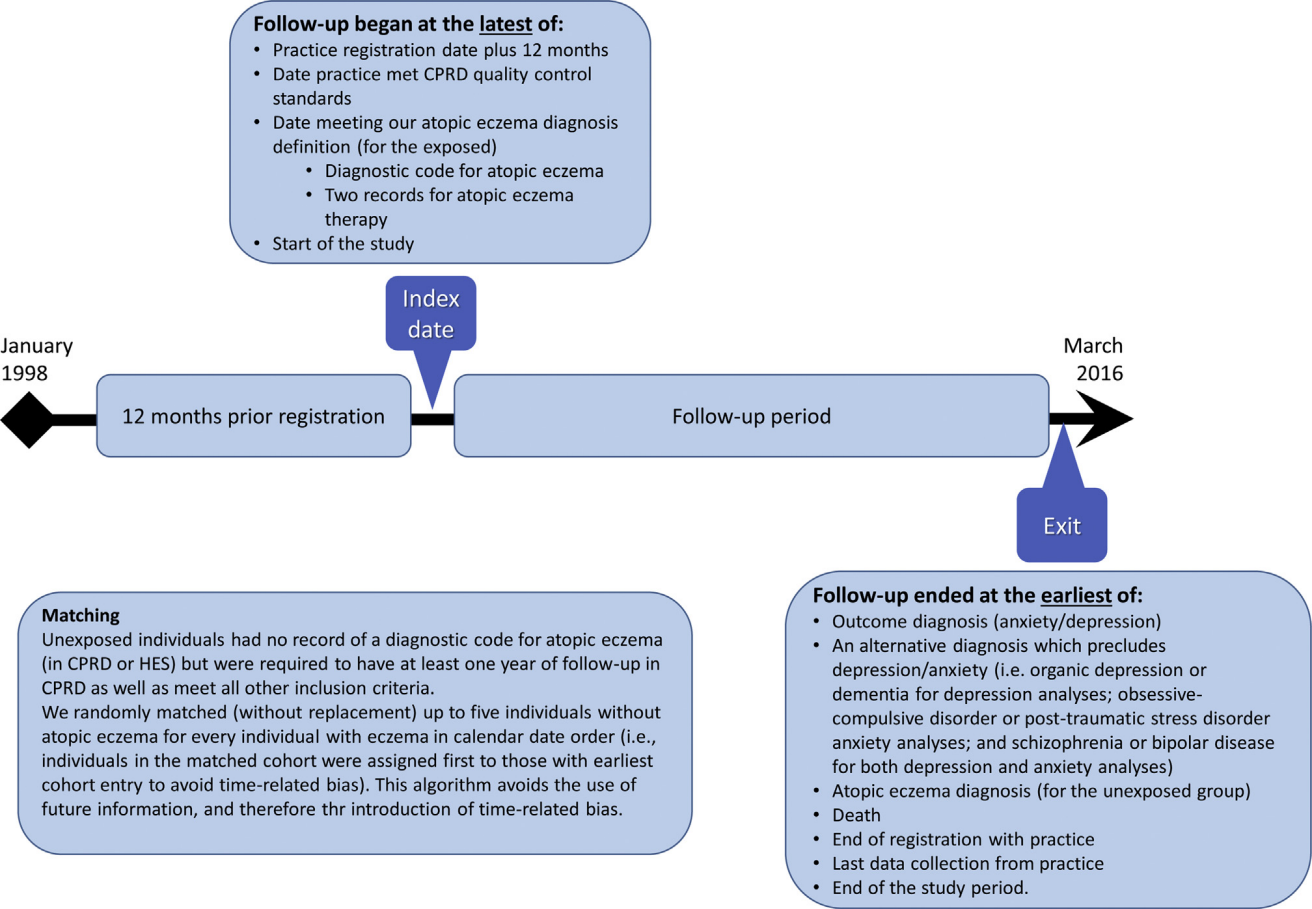
Visual representation of the cohort entry criteria and follow-up process.

Each atopic eczema–exposed individual was matched (without replacement) with up to 5 individuals without atopic eczema on sex, age, general practice, and calendar time. Unexposed individuals had no record of a diagnostic code for atopic eczema (in CPRD or HES) but were required to have at least 1 year of follow-up in CPRD as well as meet all other inclusion criteria. To minimize selection bias due to the exclusion of unmatched individuals and closely adjust for its effects, age was matched in 15-year strata and used as the underlying time scale for all analysis. To avoid misclassifying unexposed person-time, individuals could contribute unexposed person-time until the date of their first record of a diagnostic code for atopic eczema, regardless of later therapies prescribed (see Figure E1 in this article's Online Repository at www.jaci-inpractice.org).

### Outcomes

We considered depression and anxiety as separate outcomes, with onset defined as the date of the first recorded diagnosis in either CPRD or HES (any inpatient hospital diagnosis). Codes for the depression outcome were those compatible with unipolar depression,^45^ and for the anxiety outcome, included those consistent with generalized anxiety and panic disorders. We considered broader definitions of depression and anxiety in prespecified sensitivity analyses (see this article's “Code lists for the outcomes (depression and anxiety)” section in the Online Repository at www.jaci-inpractice.org).

### Defining follow-up

Individuals entered the cohort at the latest of: practice registration date plus 12 months; the date their practice met CPRD quality control standards; the date an individual met our atopic eczema diagnosis definition; or the start of the study (January 2, 1998). Individuals without atopic eczema entered the cohort on the same day as their matched atopic eczema–exposed case. We included a mandatory “wash-in” period of 12 months before cohort entry to ensure adequate time to capture true incident outcome diagnoses, as well as other baseline variables (eg, body mass index [BMI] and smoking).^46^

Cohort members were followed until the first of the following events: anxiety or depression diagnosis (depending on analysis); a diagnosis suggesting an alternative cause for each outcome (ie, organic depression or dementia for depression analyses; obsessive-compulsive disorder or post-traumatic stress disorder for anxiety analyses; and schizophrenia or bipolar disease for both depression and anxiety analyses); record of a morbidity code for an atopic eczema diagnosis (for the unexposed group); death date recorded in CPRD; end of registration with practice; last data collection from practice; or the end of the study (March 31, 2016).

### Covariates

**Figure E2:**
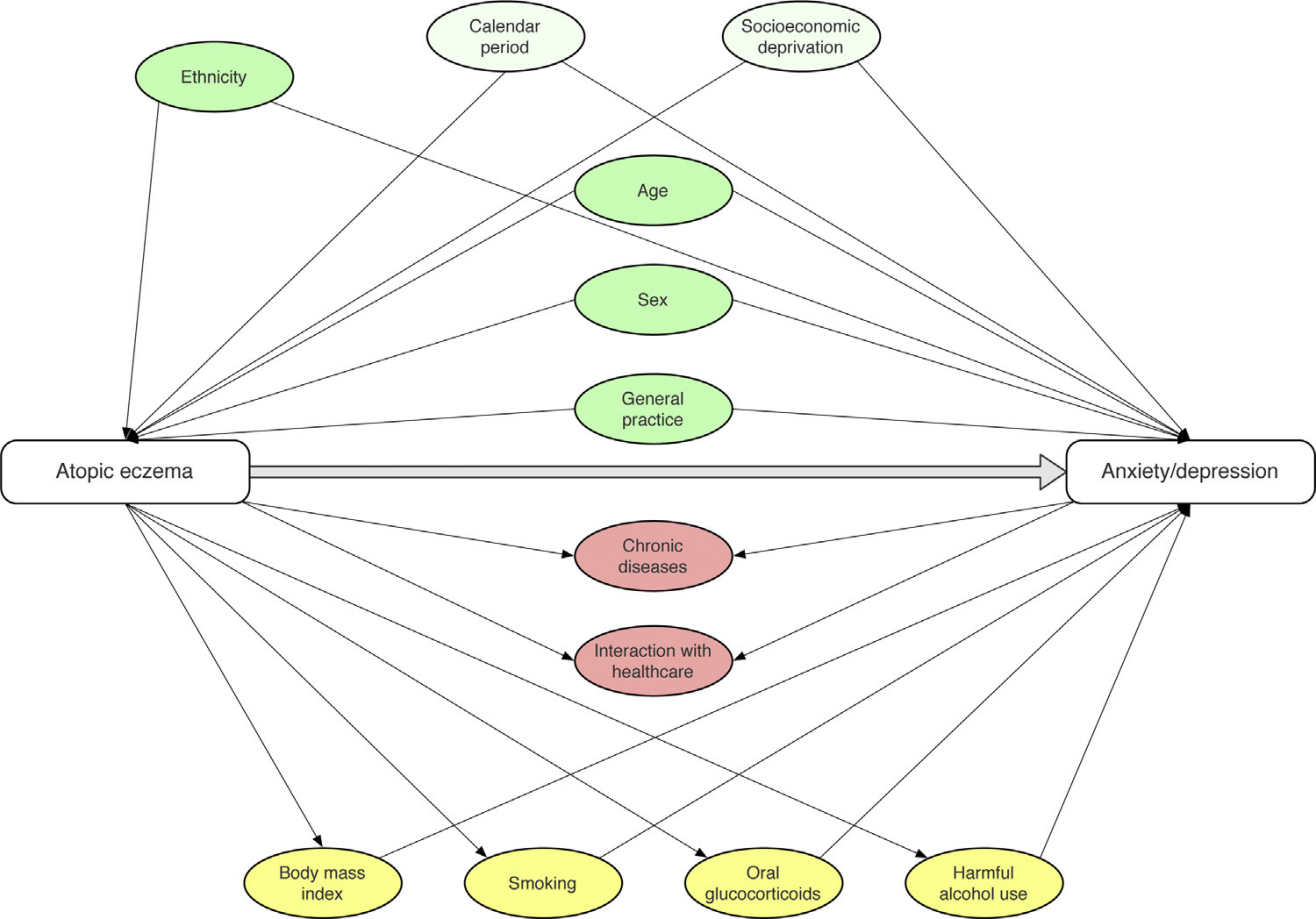
Directed acyclic graph illustrating implicitly assumed causal structure underlying our adjusted models.

Covariate selection was guided by a literature review and construction of a directed acyclic graph to avoid collider bias^47^, ^48^ (see this article's “Directed acyclic graph” section, Figure E2, and Tables E1 and E2 in the Online Repository at www.jaci-inpractice.org). Age, calendar period, sex, and level of deprivation (as quintiles of the Index of Multiple Deprivation score) and ethnic group were deemed plausibly associated with both exposure and outcome, and not on the causal pathway (ie, potential confounders). We considered BMI, smoking status, harmful alcohol use, and high-dose oral glucocorticoid as possible mediators of the association between atopic eczema and depression/anxiety. The data sources and definitions used to identify all covariates are detailed in this article's “Algorithms to identify BMI and steroid use data” and “Algorithms to identify BMI and steroid use data” sections in the Online Repository at www.jaci-inpractice.org and morbidity code lists are available to download.^49^

### Statistical analysis

We assessed the effect of the atopic eczema exposure on each outcome (depression or anxiety) using Cox regression stratified by matched set. We included the covariates used for matching in an initial crude model (implicitly adjusted for sex and general practice by stratification on matched set, and for age through the underlying timescale). We then adjusted for the remaining prespecified potential confounders (calendar period and Index of Multiple Deprivation) in an adjusted model. Finally, we also further adjusted for potential mediators of the relationship between atopic eczema and depression/anxiety (BMI; smoking; harmful alcohol and high-dose oral glucocorticoid use) in a third model. To preserve matching, analyses only included valid matched sets; that is, entire matched sets were excluded if the atopic eczema–exposed individual was excluded (because of preexisting outcome diagnosis at cohort entry, or because of missing BMI or smoking data in the models including possible mediators of the relationship between atopic eczema and depression/anxiety), or if no individuals without atopic eczema remained in the set.

The absolute incidence rates of new depression and anxiety could be directly calculated among those with atopic eczema, but matching precluded a similar approach in those without atopic eczema (because this was not a representative sample of the general population). We, therefore, estimated incidence rates in those without atopic eczema by multiplying rates in those with atopic eczema by the corresponding estimated hazard ratio (HR) (after inverting it to compare unexposed with exposed).^50^ We calculated attributable risks as the difference between the incidence rates in those with and without atopic eczema, and the population-attributable risks by using the estimated HR and assuming the prevalence of atopic eczema to be 10%.^51^

We conducted a series of sensitivity analyses to explore possible sources of bias introduced by: strict definitions of the psychiatric diagnoses; use of a “mixed” incident and prevalent cohort; differential practice attendance; or restrictive algorithm-based definitions of atopic eczema (see Table E3 in this article's Online Repository at www.jaci-inpractice.org).

In prespecified secondary analyses, we (1) redefined atopic eczema exposure using atopic eczema severity as a time-updated variable and compared incidence rates of depression and anxiety in those with mild, moderate, or severe atopic eczema to those with no atopic eczema and (2) explored possible effect modification of the relationship between atopic eczema and depression/anxiety by age, sex, and calendar period.

We checked the proportional hazards assumption for the main analysis models through visual inspection of Schöenfeld residual plots. All *P* values reported are based on likelihood-ratio tests, with 99% CI.^52^ Statistical analysis was performed using Stata, version 15.1 (StataCorp LP, College Station, Texas).

## Results

### Baseline characteristics

**Figure 1:**
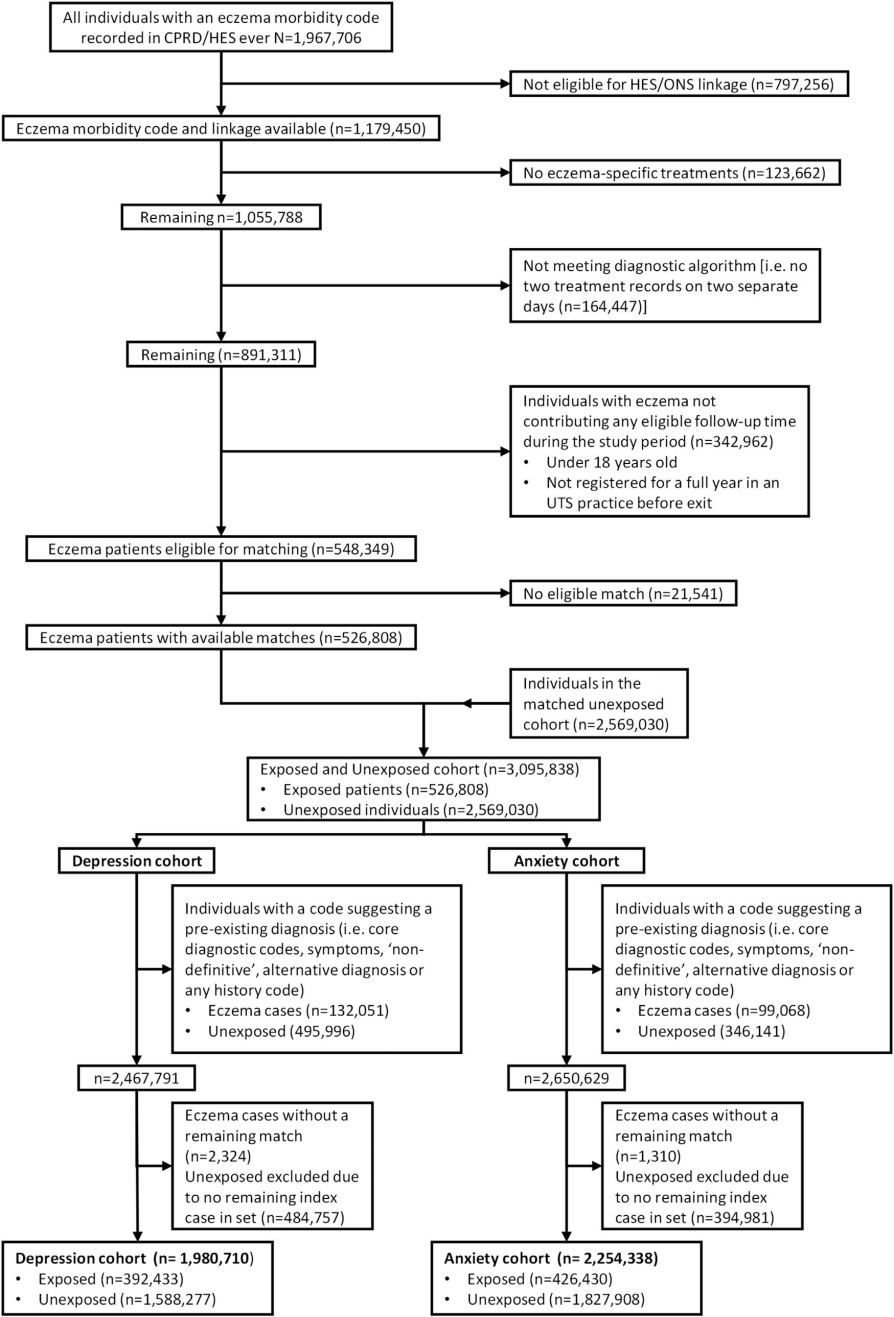
Flow diagram showing the creation of the cohort and reasons for exclusion (1998-2016). *ONS*, Office for National Statistics; *UTS*, up-to-standard.

We identified 3,095,838 adults aged 18 years or older, including 526,808 with atopic eczema, and matched them to 2,569,030 without eczema (Figure 1). Further exclusions of individuals with relevant preexisting psychiatric diagnoses on or before the start of follow-up yielded 2,467,791 participants in the cohort for analyses with depression as the outcome, and 2,650,629 with anxiety as the outcome (all belonging to “valid sets,” that is, matched sets with at least 1 exposed and 1 unexposed individual). Median follow-up was similar in both cohorts: 4.7 (interquartile range, 1.6-8.6) years for individuals with atopic eczema and 4.2 (interquartile range, 1.9-9.1) years for those without atopic eczema (Table I). The mean age of the atopic eczema–exposed individuals was 43.9 ± 21.7 years in the depression cohort and 44.1 ± 21.43 years in the anxiety cohort.

**Table I:** Characteristics of people with and without atopic eczema at cohort entry for both depression and anxiety cohorts

Characteristic∗	Depression cohort		Anxiety cohort	
	Without atopic eczema (n = 1,588,277)	With atopic eczema (n = 392,433)	Without atopic eczema (n = 1,827,908)	With atopic eczema (n = 426,430)
Follow-up (y), median (IQR)	4.21 (1.63-8.62)	4.72 (1.86-9.12)	4.18 (1.62-8.6)	4.71 (1.85-9.13)
Sex: female, n (%)	802,909 (50.6)	211,118 (53.8)	981,824 (53.1)	237,527 (55.7)
Age (y), n (%)				
18-39	828,072 (52.1)	195,455 (49.8)	941,183 (51.5)	210,764 (49.4)
40-59	355,209 (22.4)	89,126 (22.7)	431,329 (23.6)	100,592 (23.6)
≥60	404,996 (25.5)	107,852 (27.5)	455,396 (24.9)	115,074 (27.0)
Index of Multiple Deprivation (quintiles), n (%)				
1 (least deprived)	395,025 (24.9)	99,161 (25.3)	443,389 (24.3)	104,672 (24.6)
2	368,687 (23.2)	91,856 (23.4)	419,555 (23.0)	98,500 (23.1)
3	311,975 (19.6)	76,756 (19.6)	360,901 (19.7)	84,121 (19.7)
4	295,103 (18.6)	72,538 (18.5)	346,152 (18.9)	80,198 (18.8)
5 (most deprived)	217,487 (13.7)	52,122 (13.3)	257,911 (14.1)	58,939 (13.8)
BMI (kg/m^2^), mean ± SD	25.74 ± 5.1	26.01 ± 5.3	25.87 ± 5.2	26.18 ± 5.4
Normal (18.5-24.9 kg/m^2^), n (%)	574,056 (36.1)	147,216 (37.5)	663,955 (36.3)	158,315 (37.1)
Underweight (<18.5 kg/m^2^), n (%)	40,118 (2.5)	9,830 (2.5)	46,346 (2.5)	10,536 (2.5)
Overweight (25.0-29.9 kg/m^2^), n (%)	397,525 (25.0)	105,468 (26.9)	460,537 (25.2)	114,921 (27.0)
Obese (≥30.0 kg/m^2^), n (%)	209,823 (13.2)	60,643 (15.5)	258,799 (14.2)	70,714 (15.6)
Missing, n (%)	366,755 (23.1)	69,276 (17.7)	398,271 (21.8)	71,944 (16.9)
Smoking status, n (%)				
Nonsmoker	833,152 (52.5)	211,240 (53.8)	939,278 (51.4)	222,529 (52.2)
Current/ex-smoker	638,023 (40.2)	168,778 (43.0)	763,295 (41.8)	191,066 (44.8)
Missing	117,102 (7.4)	12,415 (3.2)	125,335 (6.9)	12,835 (3.0)
Harmful alcohol use, n (%)	23,244 (1.5)	7,114 (1.8)	31,639 (1.7)	9,119 (2.1)
High-dose glucocorticoids (≥20 mg/d prednisolone equivalent dose), n (%)	65,155 (4.1)	42,738 (10.9)	78,579 (4.3)	47,840 (11.2)

*IQR,* Interquartile range; *SD*, standard deviation.

∗ See this article's “Definitions for included covariates” section in the Online Repository at www.jaci-inpractice.org for details of variable definitions.

Participants with atopic eczema were less likely to have missing BMI values or smoking status, compared with those without atopic eczema, and those with missing information were more likely to be young and male (see Tables E4 and E5 in this article's Online Repository at www.jaci-inpractice.org).

### Main analysis

We explored diagnoses compatible with unipolar depression, generalized anxiety disorder, and panic disorders as the primary outcomes. There was a 1.14-fold (99% CI, 1.12-1.16) increase in the HR for depression in those with atopic eczema compared with those without, after adjusting for age, sex, general practice, current calendar period, and Index of Multiple Deprivation at cohort entry (Table II. For full model, see Table E6 in this article's Online Repository at www.jaci-inpractice.org). Atopic eczema was also associated with a 1.17-fold (99% CI, 1.14-1.19) increase in the risk of anxiety. Both estimates were attenuated after additionally adjusting for BMI, smoking status, harmful alcohol use, and high-dose glucocorticoid use (variables that may mediate the relationship between atopic eczema and depression/anxiety) (depression: HR, 1.10; 99% CI, 1.10-1.12; anxiety: HR, 1.12; 99% CI, 1.10-1.15). The absolute excess risk of depression/anxiety among those with atopic eczema that could be considered due to atopic eczema (attributable risk) was 160 per 100,000 person-years with atopic eczema (99% CI, 146-186) for depression and 144 per 100,000 for anxiety (99% CI, 115-153) although the excess risk of depression/anxiety in the population that could be considered due to atopic eczema (population-attributable risk) was 1.4% (95% CI, 1.2-1.6) for depression and 1.7% (1.4-1.9) for anxiety (see Table E7 in this article's Online Repository at www.jaci-inpractice.org) (these estimates were calculated assuming a 10% prevalence of atopic eczema and would increase if atopic eczema were more common).

**Table II:** HRs (99% CI) from Cox regression for the association between atopic eczema and anxiety and depression

Cohort	No.	Events/PYAR	Minimally adjusted, HR (99% CI)∗	Adjusted, HR (99% CI)†	Additionally adjusted for potential mediators‡		
					No.	Events/PYAR	HR (99% CI)
Depression							
No atopic eczema	1,588,277	102,882/8,935,934	1.00 (reference)	1.00 (reference)	1,054,673	76,638/6,531,745	1.00 (reference)
Atopic eczema	392,433	31,322/2,354,118	1.14 (1.12-1.16)	1.14 (1.12-1.16)	316,332	27,405/2,042,715	1.10 (1.07-1.12)
Anxiety							
No atopic eczema	1,818,796	82,137/10,187,499	1.00 (reference)	1.00 (reference)	1,237,423	63,592/7,566,056	1.00 (reference)
Atopic eczema	424,109	24,283/2,543,384	1.17 (1.14-1.19)	1.17 (1.14-1.19)	345,967	21,666/2,223,508	1.12 (1.09-1.15)

*IMD,* Index of Multiple Deprivation; *PYAR,* person-years at-risk.

All models were fitted to people with complete data for all included variables. Matched sets without at least 1 individual with atopic eczema and 1 without were excluded. HRs were estimated from a Cox regression model with current age as the underlying time scale, stratified by matched set (sex, age, and general practice).

∗ *Minimally adjusted* model accounted for the matching variables (1,980,710 participants in the depression cohort [1,920,172 unique people] and 2,242,905 in the anxiety cohort [2,171,784 unique people]).

† The *adjusted* model additionally included current calendar period (years: 1998-2001, 2002-2006, 2007-2011, and 2012-2016) and quintiles of IMD at cohort entry (same participants as in the minimally adjusted).

‡ *Additionally adjusted for potential mediators*: BMI (categorized as normal, 18.5-24.9 kg/m^2^; underweight, <18.5 kg/m^2^; overweight 25.0-29.9 kg/m^2^; and obese ≥30.0 kg/m^2^), smoking status, and alcohol and high-dose glucocorticoid use (≥20 mg/d prednisolone equivalent dose), both as time-updated variables (1,371,005 participants in the depression cohort [1,322,284 unique people] and 1,583,390 participants in the anxiety cohort [1,583,390 unique people]).

Our sensitivity analyses showed broadly similar effect estimates—those from the main analysis (Table E3).

### Secondary analyses

#### Atopic eczema severity

**Figure 2:**
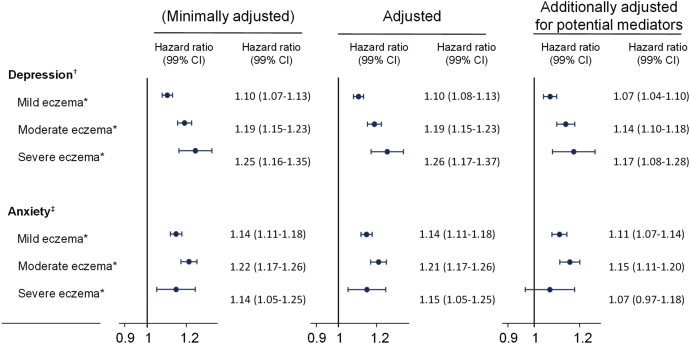
HRs (99% CI) for the association between eczema severity (time-updated) and depression and anxiety. *IMD*, Index of Multiple Deprivation. All models were fitted to people with complete data for all included variables. Sets without at least 1 exposed and 1 unexposed were excluded. HRs were estimated from a Cox regression model with current age as the underlying time scale, stratified by matched set (sex, age, and general practice). A *minimally adjusted* model accounted for the matching variables (1,980,710 participants in the depression cohort [1,920,172 unique people] and 2,242,905 in the anxiety cohort [2,171,784 unique people]). The *adjusted* model additionally included current calendar period (years: 1998-2001, 2002-2006, 2007-1201, and 2012-2016,) and quintiles of IMD at cohort entry (same participants as in the minimally adjusted). A final model, *additionally adjusted for potential mediators*, also included BMI (categorized as normal, 18.5-24.9 kg/m^2^; underweight, <18.5 kg/m^2^; overweight 25.0-29.9 kg/m^2^; obese ≥30.0 kg/m^2^), smoking status, and alcohol and high-dose corticosteroid use (≥20 mg/d prednisolone equivalent dose), both as time-updated variables (1,371,005 participants in the depression cohort [1,322,284 unique people] and 1,583,390 in the anxiety cohort [1,583,390 unique people]). *Compared with no atopic eczema. †Depression: *P* values were less than .0001 for linearity in all models, and for departure from linearity were as follows: minimally adjusted *P* = .3810; adjusted *P* = .3832; and additionally adjusted for potential mediators *P* = .6983. ‡Anxiety: *P* values were less than .0001 for linearity in all models, and less than .0001 for departure from linearity in all models.

Regardless of atopic eczema severity level, we saw evidence for an association between atopic eczema and both depression and anxiety (Figure 2). Compared with those without atopic eczema, the risk of depression increased with increasing atopic eczema severity (*P* < .0001 for linearity; *P* = .3832 for departure from linearity in the adjusted model; and *P* = .6983 for departure from linearity in the model additionally adjusted for potential mediators). However, the results of analyses exploring the relationship between atopic eczema severity and anxiety did not demonstrate a similarly clear dose-response relationship; for mild and moderate atopic eczema, there was some evidence of a similar dose-response increase, but there was strong statistical evidence for departure from linearity (*P* < .0001) (see Table E8 in this article's Online Repository at www.jaci-inpractice.org).

#### Effect modification by sex, age, and calendar period

We saw some evidence (*P* < .0001) for sex modifying the effect of atopic eczema on depression, with a slightly higher risk of depression in those with atopic eczema compared with those without in men (1.19; 99% CI, 1.16-1.23) than in women (1.11; 99% CI, 1.08-1.13). We saw a similar pattern for risk of anxiety in those with and without atopic eczema after stratifying on sex (HR [99% CI]: men, 1.22 [99% CI, 1.17-1.27]; women, 1.14 [99% CI, 1.11-1.17]; *P* = .0003 for interaction). We also saw evidence for effect modification by current age, with the HR comparing those with atopic eczema to those without for both depression (*P* < .0001) and anxiety (*P* = .0052) being higher in those aged 40 to 59 years, compared with younger and older age groups. There was no evidence of a change in the effect of atopic eczema on both depression (*P* = .3229) and anxiety (*P* = .287) in different calendar periods (see Table E9 in this article's Online Repository at www.jaci-inpractice.org).

## Discussion

### Main findings

We found that (treated) atopic eczema was associated with a 14% increase in the risk of newly diagnosed depression (adjusted HR; 99% CI, 1.12-1.16) and a 17% increase in the risk of a subsequent anxiety diagnosis (adjusted HR; 99% CI, 1.14-1.19). These associations were only slightly attenuated after further adjusting for potential mediators of the association between atopic eczema and anxiety/depression (BMI, smoking status, and alcohol and high-dose glucocorticoid use) and were present at all levels of atopic eczema disease severity. Risk of a new depression diagnosis increased linearly with increasing atopic eczema severity, providing strong evidence for a dose-response association. The outcomes were diagnoses compatible with unipolar depression, generalized anxiety disorder, and panic disorders, but we considered broader definitions of depression/anxiety in subsequent sensitivity analyses.

### Strengths and limitations

We identified a large, nationally representative sample of people, the largest reported to date,^20^, ^21^ ensuring precise effect estimations and increased generalizability. We used a validated diagnostic algorithm to identify atopic eczema in primary care,^53^ and relied on highly specific physician diagnoses rather than self-reported outcomes.^54^, ^55^, ^56^ We chose the covariates included in the analysis on the basis of *a priori* reasoning (see this article's “Directed acyclic graph” section; Figure E2).^48^ Although some chronic conditions may be associated with atopic eczema,^57^ as well as with depression/anxiety,^58^ in the context of this study, we did not consider these conditions fit the definition for confounding because the potential confounder (chronic comorbidity) could be considered to be either a consequence of the outcome (anxiety/depression), or to mediate the relationship between exposure and outcome (see this article's “Directed acyclic graph” section).

We deemed other factors (ie, BMI, smoking, systemic glucocorticoids, harmful alcohol use) as likely mediators of the effect of atopic eczema on depression and anxiety, rather than confounders; we consequently adjusted for these variables separately. Atopic eczema may be associated with the later development of conditions such as cardiovascular disease and various malignancies,^50^, ^57^ but exploring the potential mediating role of chronic comorbidity was beyond the scope of our analysis.

The study also has several limitations. The algorithm we used to define atopic eczema excluded untreated individuals, reducing its sensitivity to detect milder cases.^59^ This limitation was mitigated by the availability of primary care data, because 97% of those with atopic eczema in the United Kingdom are managed in primary care,^60^, ^61^ and by including emollients, which are routinely prescribed for atopic eczema in the United Kingdom.^62^ The results also remained robust in sensitivity analyses using less-restrictive atopic eczema definitions. Analyses stratified by atopic eczema severity provided further reassuring evidence of an association between atopic eczema and anxiety/depression even among mild cases. However, our definition of atopic eczema severity might have misclassified individuals with severe atopic eczema as having less severe disease if they refused medical therapy.^63^ Misclassification of disease status or severity may have overestimated or underestimated the real association between severity of eczema and anxiety/depression because early symptoms of depression/anxiety could influence diagnostic and treatment preferences. However, general practitioners recorded their depression/anxiety diagnoses independently and prospectively, so reverse causality likely affected all study participants equally regardless of atopic eczema status (ie, nondifferential misclassification, suggesting bias toward the null rather than a spurious association).

A further limitation of our eczema severity definition was that we were unable to capture symptom reduction or resolution (absence of a record for eczema does not necessarily mean absence in symptoms). Consequently, we considered individuals as having moderate or severe disease from the date they met the respective definition, and may therefore have wrongly classified people as having moderate/severe eczema when their symptoms had reduced or resolved. The result of wrongly classifying individuals as having more severe disease when their symptoms had actually remitted would only be to dilute the effect of eczema severity on depression/anxiety and bias our effect estimate to null.

Follow-up began in adulthood, resulting in a mixed cohort of prevalent and incident (newly diagnosed) atopic eczema cases, introducing possible bias due to left truncation (ie, the possibility of an outcome event occurring before cohort entry), with consequent underestimation or overestimation of the effect of atopic eczema on depression and anxiety. However, following only incident cases when exploring predominantly adult-onset outcomes would have shortened follow-up and limited the study's power. In addition, the exact onset date of a relapsing condition such as atopic eczema cannot be captured accurately in routinely collected data. In such circumstances, a dynamic cohort including prevalent cases is preferred.^64^ A sensitivity analysis offered evidence against bias introduced by including both “incident” and prevalent atopic eczema cases in our cohort because it showed broadly similar results in those with prevalent atopic eczema and those more likely to have new-onset atopic eczema.

Smoking status and/or BMI were not recorded for some study participants, and it is likely that whether smoking status/BMI was recorded or not was dependent on having atopic eczema or anxiety/depression (ie, missing not-at-random). BMI and smoking status are often captured opportunistically and are therefore more likely to be recorded in those who consult their general practitioner more frequently (due to health-seeking behavior or chronic conditions).^65^ Although previous studies suggested no clear-cut association between physical illness and detection of psychiatric diagnoses in primary care,^66^, ^67^ the possibility of selection bias when applying complete case analysis (ie, including only those with complete data) remains. In our study, this did not affect the main analysis, because the variables containing missing data were not included in the main adjusted analysis (they were considered as potential mediators). Comparable results from the model including smoking and BMI also provide evidence against substantial bias introduced by missing data. Finally, general practitioners do not routinely record patients' quality of sleep, and we were not able to assess the extent to which itch-related sleep disturbances mediate the development of depression and anxiety among people with atopic eczema.^30^

### Comparisons to existing literature

An association between atopic eczema, depression, and anxiety has been described in cross-sectional and case-control studies, in which the temporal sequence (ie, whether atopic eczema precedes depression or anxiety, or vice versa) could not be determined.^16^, ^17^, ^18^, ^19^, ^20^, ^21^, ^22^ The few longitudinal studies that addressed this question had inconsistent results.^32^, ^33^, ^34^ These studies were limited by short follow-up windows^34^; inclusion of selected, nonrepresentative populations (eg, male military conscripts^34^ or secondary care diagnoses^32^, ^33^); no account of atopic eczema disease severity^32^, ^34^; low-quality or no individual-level information on lifestyle variables^32^, ^34^; and reliance on disease-specific medication usage as a nonspecific proxy measure to ascertain depression and anxiety.^33^, ^34^ Notably, a recent Danish cohort study demonstrated point estimates that were in line with the estimates reported in our study, but the association was not evident in the adjusted models that included health care consumption.^33^

### Interpretation and clinical implications

Atopic eczema, like several other chronic conditions,^58^ is associated with depression/anxiety. The link to chronic mental illness further supports the view of atopic eczema as a systemic disorder.^68^ Our results suggest that the association between atopic eczema and depression/anxiety is not substantially mediated through glucocorticoid treatment, obesity, smoking, or harmful alcohol intake. Evidence against a dose-response association between atopic eczema severity and anxiety could not only imply different pathophysiological mechanisms but also reflect misclassification of outcome, because the anxiety outcome was more heterogeneously defined. Our findings suggest that atopic eczema was more strongly associated with depression and anxiety in those aged 40 to 59 years (compared with younger and older age groups). However, it is unclear why; further research could investigate possible explanations for differences in the association between atopic eczema and depression/anxiety risk in those at different ages (eg, different age-specific coping strategies, or increased health care contacts due to active cardiovascular screening in that age group). Future research could also support our findings of a dose-response association between atopic eczema and depression/anxiety by including people with more severe forms of these conditions (eg, identified using prescriptions for antidepressants and anxiolytic medications).

Although our results apply directly to UK primary care, they are likely to be relevant in other settings, especially where there is primary care–oriented universal access to health care. Mental illness is underdiagnosed in people with skin or other chronic diseases,^69^, ^70^, ^71^ but their detection and treatment might improve atopic eczema control by facilitating better adherence to skin disease treatment,^72^ or through direct anti-inflammatory actions of antidepressants.^73^ Current UK guidelines address only the management of atopic eczema in children, emphasizing the importance of assessing the psychosocial well-being and quality of life.^74^ Recent guidelines from the European Academy of Dermatology and Venereology comment briefly on the influence of psychological and emotional factors on the clinical course of atopic eczema.^5^ Neither of these guidelines mentions the long-term mental health implications of atopic eczema. Our findings suggest that depression and anxiety should be addressed explicitly in future guideline updates. Further research is needed to explore and define possible mediators; to characterize subpopulations at increased risk (eg, those with adult-onset atopic eczema, or those with more active variants of the disease); and to elucidate the feasibility and effectiveness of screening, early detection, and prevention of depression and anxiety among those with atopic eczema.

## Conclusions

Individuals affected with atopic eczema were more likely to develop depression and anxiety, regardless of atopic eczema severity. Strong evidence for a dose-response relationship between atopic eczema severity and depression supports a causal association. These results highlight the importance of a comprehensive bio-psycho-social approach to limit common mental disorders in those with atopic eczema and could guide recommendations for the management of atopic eczema.

## Figures and Tables

**Table E1:** Univariable associations between covariates and depression

Variable	Events/PYAR	Rate/100,000 PYAR	HR (99% CI)
Atopic eczema exposure			
Without atopic eczema	102,882/8,935,934	1,151 (1,142-1,161)	1.00 (reference)
With atopic eczema	31,322/2,354,118	1,331 (1,311-1,350)	1.14 (1.11-1.16)
Atopic eczema severity			
Unexposed	102,882/8,935,934	1,151 (1,142-1,161)	1.00 (reference)
Mild	19,116/1,436,377	1,331 (1,306-1,356)	1.10 (1.07-1.12)
Moderate	10,301/786,352	1,310 (1,277-1,344)	1.19 (1.15-1.23)
Severe	1,905/131,389	1,450 (1,367-1,538)	1.25 (1.16-1.35)
Sex			
Male	49,110/5,599,286	877 (867-887)	NA
Female	85,094/5,690,766	1495 (1482-1509)	NA
Age at cohort entry (y)			
18-19	24,147/1,511,015	1,598 (1,572-1,625)	1.00 (reference)
20-29	26,094/1,698,066	1,537 (1,512-1,561)	1.00 (0.88-1.14)
30-39	23,230/1,817,250	1,278 (1,257-1,300)	1.10 (0.84-1.44)
40-49	17,298/1,599,240	1,082 (1,061-1,103)	1.01 (0.65-1.57)
50-59	12,621/1,569,384	804 (786-823)	1.29 (0.71-2.36)
60-69	11,943/1,542,594	774 (756-793)	2.30 (1.16-4.56)
70-79	12,633/1,133,250	1,115 (1,090-1,141)	3.50 (1.69-7.25)
≥80	6,238/419,254	1,488 (1,440-1,537)	3.95 (1.82-8.59)
Calendar period (y)			
1998-2001	22,063/1,422,827	1,551 (1,524-1,578)	1.00 (reference)
2002-2006	37,260/2,937,645	1,268 (1,252-1,285)	0.75 (0.64-0.87)
2007-2011	42,449/3,973,737	1,068 (1,055-1,082)	0.59 (0.49-0.71)
2012-2016	32,432/2,955,843	1,097 (1,082-1,113)	0.56 (0.46-0.69)
IMD (quintiles)			
1 (least deprived)	28,280/2,942,268	961 (947-976)	1.00 (reference)
2	28,468/2,687,862	1,059 (1,043-1,075)	1.11 (1.08-1.14)
3	26,471/2,229,329	1,187 (1,169-1,206)	1.22 (1.19-1.26)
4	27,669/1,995,052	1,387 (1,366-1,409)	1.46 (1.41-1.51)
5 (most deprived)	23,316/1,435,541	1,624 (1,597-1,652)	1.72 (1.66-1.79)
BMI (kg/m^2^), mean ± SD			
Normal (18.5-24.9 kg/m^2^)	3,613 ± 206,460	1,750 (1,677-1,827)	1.00 (reference)
Underweight (<18.5 kg/m^2^)	47,272 ± 3,898,062	1,213 (1,198-1,227)	0.85 (0.80-0.90)
Overweight (25.0-29.9 kg/m^2^)	32,541 ± 2,989,894	1,088 (1,073-1,104)	0.89 (0.84-0.94)
Obese (≥30.0 kg/m^2^)	21,096 ± 1,521,982	1,386 (1,362-1,411)	1.08 (1.01-1.14)
Smoking status			
Nonsmoker	61,985/6,112,858	1,014 (1,004-1,025)	1.00 (reference)
Current/ex-smoker	66,260/4,456,536	1,487 (1,472-1,502)	1.60 (1.57-1.63)
Harmful alcohol use∗			
No	129,457/11,080,950	1,168 (1,160-1,177)	1.00 (reference)
Yes	4,747/209,102	2,270 (2,187-2,357)	2.57 (2.43-2.71)
High-dose glucocorticoids∗ (≥20 mg/d prednisolone equivalent dose)			
No	132,592/11,219,476	1,182 (1,173-1,190)	1.00 (reference)
Yes	1,612/70,576	2,284 (2,142-2,435)	2.10 (1.92-2.29)

*NA,* Not applicable/available; *PYAR,* person-years at-risk.

Univariable HRs are derived from Cox regression models with current age as the underlying timescale, stratified by matched set (sex, age, and general practice). Models were fitted to patients with complete data who are included in valid sets (ie, with at least 1 exposed and 1 unexposed individual).

∗ Measured as a time-updated variable.

**Table E2:** Univariable associations between covariates and anxiety

Variable	Events/PYAR	Rate/100,000 PYAR	HR (99% CI)
Atopic eczema exposure			
Without atopic eczema	82,137/10,187,499	806 (799-814)	1.00 (reference)
With atopic eczema	24,283/2,543,384	955 (939-971)	1.17 (1.14-1.19)
Atopic eczema severity			
Unexposed	82,137/10,187,499	806 (799-814)	1.00 (reference)
Mild	15,093/1,543,672	978 (957-998)	1.14 (1.11-1.17)
Moderate	7,822/853,452	917 (890-944)	1.21 (1.17-1.26)
Severe	1,368/146,259	935 (872-1003)	1.14 (1.04-1.25)
Sex			
Male	32,586/5,928,234	550 (542-558)	NA
Female	73,834/6,802,649	1,085 (1,075-1,096)	NA
Age at cohort entry (y)			
18-19	18,218/1,582,297	1,151 (1,130-1,174)	1.00 (reference)
20-29	22,028/1,950,021	1,130 (1,110-1,149)	0.80 (0.69-0.92)
30-39	20,658/217,9519	948 (931-965)	0.66 (0.51-0.86)
40-49	14,555/1,892,615	769 (753-786)	0.66 (0.44-0.99)
50-59	10,918/1,779,862	613 (598-629)	0.51 (0.29-0.90)
60-69	9,436/1,668,280	566 (551-581)	0.62 (0.32-1.19)
70-79	7,603/1,210,115	628 (610-647)	0.72 (0.35-1.48)
≥80	3,004/468,173	642 (612-673)	0.86 (0.39-1.93)
Calendar period (y)			
1998-2001	16,323/1,572,503	1,038 (1,017-1,059)	1.00 (reference)
2002-2006	28,605/3,294,860	868 (855-881)	0.96 (0.80-1.15)
2007-2011	32,291/4,494,729	718 (708-729)	0.83 (0.67-1.03)
2012-2016	29,201/3,368,791	867 (854-880)	0.87 (0.69-1.10)
IMD (quintiles)			
1 (least deprived)	23,246/3,239,958	717 (705-730)	1.00 (reference)
2	23,013/2,989,450	770 (757-783)	1.09 (1.05-1.22)
3	20,698/2,535,226	816 (802-831)	1.15 (1.11-1.19)
4	21,398/2,296,647	932 (915-948)	1.30 (1.25-1.35)
5 (most deprived)	18,065/1,669,602	1,082 (1,061-1,103)	1.42 (1.36-1.48)
BMI (kg/m^2^), mean ± SD			
Normal (18.5-24.9 kg/m^2^)	3,109 ± 236,632	1,314 (1,255-1,376)	1.00 (reference)
Underweight (<18.5 kg/m^2^)	40,808 ± 4,412,253	925 (913-937)	0.86 (0.81-0.91)
Overweight (25.0-29.9 kg/m^2^)	25,695 ± 3,365,057	764 (751-776)	0.85 (0.80-0.90)
Obese (≥30.0 kg/m^2^)	16,016 ± 1,823,430	878 (861-896)	0.90 (0.84-0.96)
Smoking status			
Nonsmoker	51,195/6,778,933	755 (747-764)	1.00 (reference)
Current/ex-smoker	51,622/5,186,043	995 (984-1007)	1.44 (1.42-1.47)
Harmful alcohol use∗			
No	102,616/12,465,556	823 (817-830)	1.00 (reference)
Yes	3,804/265,326	1,434 (1,375-1,495)	2.32 (2.18-2.46)
High-dose glucocorticoids∗ (≥20 mg/d prednisolone equivalent dose)			
No	105,057/12,646,811	831 (824-837)	1.00 (reference)
Yes	1,363/84,071	1,621 (1,512-1,738)	2.12 (1.92-2.34)

*NA,* Not applicable/available; *PYAR,* person-years at-risk.

Univariable HRs are derived from Cox regression models with current age as the underlying timescale, stratified by matched set (sex, age, and general practice). Models were fitted to patients with complete data who are included in valid sets (ie, with at least 1 exposed and 1 unexposed individual).

∗ Measured as a time-updated variable.

**Table E3:** Description, justification, and summary results of sensitivity analyses

Analysis	Description	Justification	Depression			Anxiety		
			No.	Events/PYAR	Adjusted HR∗ (99% CI)	No.	Events/PYAR	Adjusted HR∗ (99% CI)
Main analysis			1,980,710	134,204/11,290,052	1.14 (1.12-1.16)	2,242,905	106,420/12,730,883	1.17 (1.14-1.19)
Sensitivity analysis 1	Repeating the primary analysis using progressively less-strict definitions of psychiatric diagnoses	To explore potential bias introduced by low sensitivity to detect psychiatric diagnoses in electronic health records, as well as by general practitioner's use of symptom codes, instead of diagnostic codes						
	(1a) Initially including symptom codes in the definitions of outcomes		1,980,710	211,534/10,970,276	1.16 (1.14-1.17)	2,242,905	175,874/12,420,852	1.18 (1.16-1.20)
	(1b) Subsequently also adding “nondefinitive” diagnostic codes		1,980,710	227,393/10,908,249	1.15 (1.14-1.17)	2,242,905	202,679/12,353,235	1.18 (1.16-1.20)
Sensitivity analysis 2	Repeating the primary analysis separately for prevalent and incident atopic eczema cases. Stratifying the analysis on the time since the initial diagnosis (0-4 or ≥5 y)	To separate “true prevalent” cases from likely incident atopic eczema cases to explore possible bias due to the choice of a “prevalent” cohort design						
	(2a) “Incident” cohort (2b) “Prevalent” cohort		1,431,318 549,392	97,372/8,445,494 36,832/2,844,558	1.17 (1.15-1.20) 1.05 (1.01-1.09)	1,646,703 596,202	77,545/9,614,471 28,875/3,116,412	1.19 (1.16-1.22) 1.10 (1.06-1.15)
Sensitivity analysis 3	Repeating the primary analysis including only those who consulted their general practitioner in the year before cohort entry	To explore potential bias due to differential recording of exposure, covariates, and outcomes among practice attenders and nonattenders. Robust effect implies insensitivity to bias introduced by varying degrees of health care contact	1,825,694	125,472/10,460,654	1.16 (1.14-1.18)	2,086,308	100,225/11,882,522	1.19 (1.16-1.21)
Sensitivity analysis 4	Repeating the primary analysis on redefined cohorts with a less-restrictive atopic eczema definition: atopic eczema diagnosis was ascertained using only atopic eczema diagnostic codes, with no requirement for a therapeutic code	To explore the sensitivity of the results to the definition of atopic eczema (eg, those with childhood atopic eczema may have been erroneously excluded from the primary analysis if they switched practice in adulthood, and did not require further treatments)	2,514,107	173,793/14,708,229	1.07 (1.05-1.09)	2,838,141	135,719/16,515,260	1.10 (1.08-1.12)
Sensitivity analysis 5	Repeating the primary analysis on redefined cohorts with a less-restrictive definition for those without atopic eczema (unexposed): individuals with an atopic eczema diagnosis but without 2 further eczema treatments were considered not to have atopic eczema, and could therefore be included in the pool of unexposed participants. The cohort of patients with atopic eczema remained the same (ie, eczema was defined as having at least 1 diagnostic code and 2 treatment codes)	To explore the sensitivity of the results to the definition of atopic eczema	2,002,613	135,552/11,329,039	1.14 (1.12-1.16)	2,267,537	107,660/12,771,273	1.16 (1.14-1.19)
Sensitivity analysis 6	Additionally adjusting for ethnicity (white, South Asian, black, other, or mixed, identified from CPRD and HES data). Analysis was restricted to those registered in 2006 or later, because ethnicity recording before 2006 is selective, and of low quality^E62^	To examine whether the omission of ethnicity from the primary analysis may have introduced bias, because reliable ethnicity data exists only for that period	276,853	8,251/649,041	1.16 (1.06-1.26)	340,161	8,481/80,4170	1.29 (1.18-1.40)

*PYAR*, Person-years at-risk.

All models were fitted to patients with complete data for all included variables. Sets without at least 1 exposed and 1 unexposed were excluded. HRs were estimated from a Cox regression model with current age as the underlying timescale, stratified by matched set (sex, age, and general practice), and adjusted for current calendar period (years: 1998-2001, 2002-2006, 2007-2011, and 2012-2016,) and quintiles of IMD at cohort entry.

∗ All HRs are for the outcome (ie, depression/anxiety) among those with atopic eczema, compared with those without atopic eczema.

**Table E4:** Exploring missing data—distribution of baseline characteristics in depression study population (overall, those with complete data, those with missing BMI, and those with missing smoking status)

Atopic eczema status, n (%)	Overall depression sample (n = 1,980,710 [100%])		Mediation model sample∗ (n = 1,371,005 [69.2%])		Individuals with missing BMI (n = 436,031 [22.0%])		Individuals with missing smoking status (n = 129,517 [6.5%])	
	Without atopic eczema (n = 1,588,277 [100%])	With atopic eczema (n = 392,433 [100%])	Without atopic eczema (n = 1,054,673 [66.4%])	With atopic eczema (n = 316,332 [80.6%])	Without atopic eczema (n = 366,755 [23.1%])	With atopic eczema (n = 69,276 [17.65%])	Without atopic eczema (n = 117,102 [7.4%])	With atopic eczema (n = 12,415 [3.2%])
Follow-up (y), median (IQR)	4.21 (1.63-8.62)	4.72 (1.86-9.12)	4.91 (1.95-9.49)	5.26 (2.18-9.88)	2.80 (1.13-6.18)	2.75 (1.14-5.86)	2.30 (0.86-5.23)	1.75 (0.75-3.96)
Sex: female, n (%)	802,909 (50.55)	211,118 (53.80)	593,302 (56.25)	182,005 (57.54)	136,354 (37.18)	25,982 (37.51)	40,402 (34.50)	4,446 (35.81)
Age (y), n (%)								
18-19	268,216 (16.89)	74,303 (18.93)	71,409 (6.77)	33,343 (10.54)	151,841 (41.40)	38,048 (54.92)	42,890 (36.63)	6,906 (55.63)
20-29	314,643 (19.81)	64,699 (16.49)	183,603 (17.41)	51,753 (16.36)	78,605 (21.43)	11,823 (17.07)	25,088 (21.42)	1,869 (15.05)
30-39	245,213 (15.44)	56,453 (14.39)	182,352 (17.29)	50,871 (16.08)	39,497 (10.77)	5,087 (7.34)	13,467 (11.50)	802 (6.46)
40-49	181,584 (11.43)	46,172 (11.77)	146,905 (13.93)	42,742 (13.51)	23,994 (6.54)	3,108 (4.49)	8,836 (7.55)	417 (3.36)
50-59	173,625 (10.93)	42,954 (10.95)	147,029 (13.94)	40,500 (12.80)	18,447 (5.03)	2,179 (3.15)	7,006 (5.98)	300 (2.42)
60-69	177,634 (11.18)	44,460 (11.33)	154,768 (14.67)	42,306 (13.37)	15,287 (4.17)	1,903 (2.75)	5,518 (4.71)	286 (2.30)
70-79	143,603 (9.04)	39,351 (10.03)	117,080 (11.10)	36,146 (11.43)	16,782 (4.58)	2,692 (3.89)	6,094 (5.20)	575 (4.63)
≥80	83,759 (5.27)	24,041 (6.13)	51,527 (4.89)	18,671 (5.90)	22,302 (6.08)	4,436 (6.40)	8,203 (7.01)	1,260 (10.15)
IMD (quintiles), n (%)								
1 (least deprived)	395,025 (24.87)	99,161 (25.27)	266,815 (25.30)	80,001 (25.29)	88,989 (24.26)	17,547 (25.33)	28,477 (24.32)	3,164 (25.49)
2	368,687 (23.21)	91,856 (23.41)	247,499 (23.47)	74,522 (23.56)	83,470 (22.76)	15,846 (22.87)	25,338 (21.64)	2,706 (21.80)
3	311,975 (19.64)	76,756 (19.56)	206,425 (19.57)	61,540 (19.45)	71,649 (19.54)	13,673 (19.74)	22,828 (19.49)	2,656 (21.39)
4	295,103 (18.58)	72,538 (18.48)	194,459 (18.44)	58,471 (18.48)	68,535 (18.69)	12,819 (18.50)	22,398 (19.13)	2,270 (18.28)
5 (most deprived)	217,487 (13.69)	52,122 (13.28)	139,475 (13.22)	41,798 (13.21)	54,112 (14.75)	9,391 (13.56)	18,061 (15.42)	1,619 (13.04)
BMI (kg/m^2^), mean ± SD	25.74 ± 5.08	26.01 ± 5.25	25.87 ± 5.06	26.03 ± 5.24	NA	NA	24.78 ± 5.60	25.63 ± 5.89
Normal (18.5-24.9 kg/m^2^), n (%)	574,056 (36.14)	147,216 (37.51)	30,884 (2.93)	9,320 (2.95)	NA	NA	610 (0.52)	91 (0.73)
Underweight (<18.5 kg/m^2^)	40,118 (2.53)	9,830 (2.50)	486,260 (46.11)	143,793 (45.46)	NA	NA	3,829 (3.27)	513 (4.13)
Overweight (25.0-29.9 kg/m^2^)	397,525 (25.03)	105,468 (26.88)	351,348 (33.31)	103,722 (32.79)	NA	NA	1,932 (1.65)	332 (2.67)
Obese (≥30.0 kg/m^2^)	209,823 (13.21)	60,643 (15.45)	186,181 (17.65)	59,497 (18.81)	NA	NA	1,106 (0.94)	239 (1.93)
Missing	366,755 (23.09)	69,276 (17.65)	NA	NA	NA	NA	109,625 (93.61)	11,240 (90.54)
Smoking status, n (%)								
Nonsmoker	833,152 (52.46)	211,240 (53.83)	576,625 (54.67)	169,241 (53.50)	163,523 (44.59)	38,553 (55.65)	NA	NA
Current/ex-smoker	638,023 (40.17)	168,778 (43.01)	478,048 (45.33)	147,091 (46.50)	93,607 (25.52)	19,483 (28.12)	NA	NA
Missing	117,102 (7.37)	12,415 (3.16)	NA	NA	109,625 (29.89)	11,240 (16.22)	NA	NA
Harmful alcohol use, n (%)†	23,244 (1.46)	7,114 (1.81)	18,235 (1.73)	6,437 (2.03)	2,729 (0.74)	583 (0.84)	546 (0.47)	68 (0.55)
High-dose glucocorticoids, n (%)†^,^‡	65,155 (4.10)	42,738 (10.89)	48,539 (4.60)	35,368 (11.18)	9,732 (2.65)	6,323 (9.13)	1,712 (1.46)	776 (6.25)

*IQR,* Interquartile range; *NA,* not applicable/available.

∗ Individuals with complete data on BMI and smoking status, belonging to a valid set (ie, a set with at least 1 exposed and 1 unexposed individual).

† Status recorded at or before cohort entry.

‡ 20 mg/d prednisolone equivalent dose.

**Table E5:** Exploring missing data—distribution of baseline characteristics in anxiety study population (overall, those with complete data, those with missing BMI, and those with missing smoking status)

Atopic eczema status, n (%)	Overall anxiety sample (n = 2,254,338 [100%])		Included in the model additionally adjusted for potential mediators∗ (n = 1,592,373 [70.1%])		Individuals with missing BMI (n = 470,215 [20.9%])		Individuals with missing smoking status (n = 138,170 [6.1%])	
	Without atopic eczema (n = 1,827,908)	With atopic eczema (n = 426,430)	Without atopic eczema (n = 1,244,303 [68.1%])	With atopic eczema (n = 348,070 [81.6%])	Without atopic eczema (n = 398,271 [21.8%])	With atopic eczema (n = 71,944 [16.9%])	Without atopic eczema (n = 125,335 [6.9%])	With atopic eczema (n = 12,835 [3.0%])
Follow-up (y), median (IQR)	4.18 (1.62-8.57)	4.71 (1.85-9.13)	4.80 (1.91-9.38)	5.21 (2.15-9.85)	2.78 (1.11-6.15)	2.75 (1.14-5.87)	2.28 (0.85-5.16)	1.75 (0.75-3.95)
Sex: female, n (%)	981,824 (53.71)	237,527 (55.70)	737,936 (59.31)	206,628 (59.36)	157,984 (39.67)	28,048 (38.99)	45,792 (36.54)	4,729 (36.84)
Age (y), n (%)								
18-19	278,370 (15.23)	75,587 (17.73)	77,429 (6.22)	34,665 (9.96)	154,202 (38.72)	38,118 (52.98)	43,453 (34.67)	6,893 (53.70)
20-29	363,408 (19.88)	71,126 (16.68)	219,418 (17.63)	57,707 (16.58)	86,304 (21.67)	12,401 (17.24)	26,926 (21.48)	1,945 (15.15)
30-39	299,405 (16.38)	64,051 (15.02)	226,984 (18.24)	58,096 (16.69)	45,625 (11.46)	5,534 (7.69)	15,000 (11.97)	864 (6.73)
40-49	224,547 (12.28)	52,644 (12.35)	183,675 (14.76)	48,993 (14.08)	28,124 (7.06)	3,408 (4.74)	9,839 (7.85)	449 (3.50)
50-59	206,782 (11.31)	47,948 (11.24)	176,065 (14.15)	45,339 (13.03)	21,176 (5.32)	2,384 (3.31)	7,738 (6.17)	327 (2.55)
60-69	199,628 (10.92)	47,523 (11.14)	174,165 (14.00)	45,245 (13.00)	16,891 (4.24)	2,042 (2.84)	6,024 (4.81)	303 (2.36)
70-79	157,992 (8.64)	41,379 (9.70)	127,811 (10.27)	37,861 (10.88)	18,891 (4.74)	3,002 (4.17)	6,792 (5.42)	652 (5.08)
≥80	97,776 (5.35)	26,172 (6.14)	58,756 (4.72)	20,164 (5.79)	27,058 (6.79)	5,055 (7.03)	9,563 (7.63)	1,402 (10.92)
IMD (quintiles), n (%)								
1 (least deprived)	443,389 (24.26)	104,672 (24.55)	305,648 (24.56)	85,271 (24.50)	95,192 (23.90)	17,897 (24.88)	29,969 (23.91)	3,203 (24.96)
2	419,555 (22.95)	98,500 (23.10)	287,847 (23.13)	80,701 (23.19)	90,126 (22.63)	16,398 (22.79)	26,988 (21.53)	2,797 (21.79)
3	360,901 (19.74)	84,121 (19.73)	244,871 (19.68)	68,311 (19.63)	78,086 (19.61)	14,301 (19.88)	24,583 (19.61)	2,776 (21.63)
4	346,152 (18.94)	80,198 (18.81)	234,775 (18.87)	65,633 (18.86)	75,275 (18.90)	13,414 (18.65)	24,299 (19.39)	2,347 (18.29)
5 (most deprived)	257,911 (14.11)	58,939 (13.82)	171,162 (13.76)	48,154 (13.83)	59,592 (14.96)	9,934 (13.81)	19,496 (15.56)	1,712 (13.34)
BMI (kg/m^2^), mean ± SD	25.87 ± 5.22	26.18 ± 5.41	25.99 ± 5.21	26.20 ± 5.40	NA	NA	24.90 ± 5.65	25.70 ± 5.97
Normal (18.5-24.9 kg/m^2^), n (%)	663,955 (36.32)	158,315 (37.13)	36,355 (2.92)	10,070 (2.89)	NA	NA	671 (0.54)	103 (0.80)
Underweight (<18.5 kg/m^2^), n (%)	46,346 (2.54)	10,536 (2.47)	567,834 (45.63)	155,158 (44.58)	NA	NA	4,277 (3.41)	541 (4.22)
Overweight (25.0-29.9 kg/m^2^), n (%)	460,537 (25.19)	114,921 (26.95)	408,820 (32.86)	113,265 (32.54)	NA	NA	2,230 (1.78)	355 (2.77)
Obese (≥30.0 kg/m^2^), n (%)	258,799 (14.16)	70,714 (15.58)	231,294 (18.59)	69,577 (19.99)	NA	NA	1,300 (1.04)	268 (2.09)
Missing, n (%)	398,271 (21.79)	71,944 (16.87)	NA	NA	NA	NA	116,857 (93.24)	11,568 (90.13)
Smoking status, n (%)								
Nonsmoker	939,278 (51.39)	222,529 (52.18)	663,686 (53.34)	180,172 (51.76)	175,018 (43.94)	39,251 (54.56)	NA	NA
Current/ex-smoker	763,295 (41.76)	191,066 (44.81)	580,617 (46.66)	167,898 (48.24)	106,396 (26.71)	21,125 (29.36)	NA	NA
Missing	125,335 (6.86)	12,835 (3.01)	NA	NA	116,857 (29.34)	11,568 (16.08)	NA	NA
Harmful alcohol use†	31,639 (1.73)	9,119 (2.14)	24,994 (2.01)	8,222 (2.36)	3,740 (0.94)	791 (1.10)	697 (0.56)	107 (0.83)
High-dose glucocorticoids†^,^‡	78,579 (4.30)	47,840 (11.22)	59,842 (4.81)	40,269 (11.57)	10,773 (2.70)	6,598 (9.17)	1,908 (1.52)	820 (6.39)

*IQR*, Interquartile range; NA, not applicable/available.

∗ Individuals with complete data on BMI and smoking status, belonging to a valid set (ie, a set with at least 1 exposed and 1 unexposed individual).

† Status recorded at or before cohort entry.

‡ ≥20 mg/d prednisolone equivalent dose.

**Table E6:** Main analyses—full models for the association between all included variables and anxiety/depression

Included variable	Depression			Anxiety		
	Minimally adjusted HR (99% CI)	Adjusted HR (99% CI)	Additionally adjusted for potential mediators HR (99% CI)	Minimally adjusted HR (99% CI)	Adjusted HR (99% CI)	Additionally adjusted for potential mediators HR (99% CI)
Atopic eczema						
Without atopic eczema	1.00 (reference)	1.00 (reference)	1.00 (reference)	1.00 (reference)	1.00 (reference)	1.00 (reference)
With atopic eczema	1.14 (1.12-1.16)	1.14 (1.12-1.16)	1.10 (1.07-1.12)	1.17 (1.14-1.19)	1.17 (1.14-1.19)	1.12 (1.09-1.15)
Calendar period (y)						
1998-2001	NA	1.00 (reference)	1.00 (reference)	NA	1.00 (reference)	1.00 (reference)
2002-2006	NA	0.74 (0.64-0.86)	0.76 (0.64-0.91)	NA	0.95 (0.79-1.14)	0.95 (0.7-1.16)
2007-2011	NA	0.59 (0.49-0.71)	0.63 (0.51-0.77)	NA	0.82 (0.67-1.02)	0.81 (0.64-1.02)
2012-2016	NA	0.55 (0.45-0.68)	0.58 (0.46-0.73)	NA	0.86 (0.68-1.08)	0.84 (0.65-1.08)
IMD (quintiles)						
1 (least deprived)	NA	1.00 (reference)	1.00 (reference)	NA	1.00 (reference)	1.00 (reference)
2	NA	1.11 (1.08-1.14)	1.08 (1.04-1.12)	NA	1.09 (0.05-1.12)	1.06 (1.02-1.10)
3	NA	1.22 (1.19-1.26)	1.16 (1.12-1.21)	NA	1.15 (0.11-1.19)	1.11 (1.07-1.16)
4	NA	1.46 (1.41-1.51)	1.34 (1.29-1.40)	NA	1.30 (1.25-1.35)	1.23 (1.18-1.28)
5 (most deprived)	NA	1.72 (1.66-1.79)	1.53 (1.46-1.60)	NA	1.42 (1.36-1.48)	1.31 (1.25-1.37)
BMI (kg/m^2^)						
Normal	NA	NA	1.00 (reference)	NA	NA	1.00 (reference)
Underweight	NA	NA	0.87 (0.81-0.92)	NA	NA	0.88 (0.82-0.93)
Overweight	NA	NA	0.91 (0.86-0.97)	NA	NA	0.87 (0.81-0.93)
Obese	NA	NA	1.08 (1.02-1.15)	NA	NA	0.91 (0.85-0.97)
Smoking status						
Nonsmoker	NA	NA	1.00 (reference)	NA	NA	1.00 (reference)
Current/ex-smoker	NA	NA	1.47 (1.44-1.51)	NA	NA	1.36 (1.33-1.39)
Harmful alcohol use						
No	NA	NA	1.00 (reference)	NA	NA	1.00 (reference)
Yes	NA	NA	2.09 (1.96-2.22)	NA	NA	1.99 (1.86-2.13)
High-dose glucocorticoids						
No	NA	NA	1.00 (reference)	NA	NA	1.00 (reference)
Yes	NA	NA	1.84 (1.69-2.07)	NA	NA	1.94 (1.75-2.16)

*NA,* Not applicable/available.

All models were fitted to patients with complete data for all included variables. Sets without at least 1 exposed and 1 unexposed individual were excluded. HRs were estimated from a Cox regression model with current age as the underlying timescale, stratified by matched set (sex, age, and general practice).

*Minimally adjusted*: N = 1,980,710 (1,920,172 unique people) in the depression cohort and N = 2,242,905 (2,171,784 unique people) in the anxiety cohort.

*Adjusted* for current calendar period and IMD at cohort entry (same participants as in minimally adjusted model).

Additionally *adjusted for potential mediators*: BMI (categorized as normal, 18.5-24.9 kg/m^2^; underweight, <18.5 kg/m^2^; overweight 25.0-29.9 kg/m^2^; and obese ≥30.0 kg/m^2^), smoking status, and alcohol and high-dose corticosteroid use (≥20 mg/d prednisolone equivalent dose), as time-updated variables. N = 1,371,005 individuals (1,322,284 unique people) in the depression cohort and N = 1,583,390 (1,583,390 unique people) in the anxiety cohort.

**Table E7:** Absolute incidence rates, incidence rate differences (attributable risks), and population-attributable risks of depression and anxiety

Cohort	Estimated incidence rate (per 100,00 PYAR) in people with atopic eczema	HR comparing rate of depression/anxiety in those with to those without atopic eczema (99% CI)∗	Inverse HR (99% CI)†	Estimated incidence rate (per 100,000 PYAR) (99% CI) of depression/anxiety in people without atopic eczema	Estimated incidence rate difference (per 100,000 PYAR) (99% CI)	Estimated population- attributable risk (%) (99% CI)‡
Depression	1331	1.14 (1.12-1.16)	0.88 (0.86-0.89)	1171 (1145-1185)	160 (146-186)	1.4 (1.2-1.6)
Anxiety	955	1.17 (1.14 to 1.19)	0.85 (0.84-0.88)	811 (802-840)	144 (115-153)	1.7 (1.4-1.9)

*PYAR,* Person-years at-risk.

∗ Adjusted for current calendar period (years: 1998-2001, 2002-06, 2007-11, and 2012-16) and quintiles of IMD at cohort entry.

† Comparing people without atopic eczema to people with atopic eczema.

‡ Estimated as *P*(HR − 1)/(1 + *P*(HR − 1)) where *P*, the prevalence of atopic eczema, is assumed to be 10% and HR is the estimated HR.

**TABLE E8:** Association between atopic eczema and anxiety/depression, by severity of atopic eczema vs no atopic eczema

Cohort/exposure	No.	Events/PYAR	Minimally adjusted HR (99% CI)	Adjusted HR (99% CI)	Additionally adjusted for potential mediators HR (99% CI)
Depression∗					
Without atopic eczema	1,588,277	102,882/8,935,934	1.00 (reference)	1.00 (reference)	1.00 (reference)
With atopic eczema					
Mild	287,944	19,116/1,436,377	1.10 (1.07-1.13)	1.10 (1.08-1.13)	1.07 (1.04-1.10)
Moderate	135,485	10,301/786,352	1.19 (1.15-1.23)	1.19 (1.15-1.23)	1.14 (1.10-1.18)
Severe	24,777	1,905/131,389	1.25 (1.16-1.35)	1.26 (1.17-1.37)	1.17 (1.08-1.28)
Anxiety†					
Without atopic eczema	1,818,796	82,137/10,187,499	1.00 (reference)	1.00 (reference)	1.00 (reference)
With atopic eczema					
Mild	310,205	15,093/1,543,672	1.14 (1.11-1.18)	1.14 (1.11-1.18)	1.11 (1.07-1.14)
Moderate	147,261	7,822/853,452	1.22 (1.17-1.26)	1.21 (1.17-1.26)	1.15 (1.11-1.20)
Severe	27,538	1,368/146,259	1.14 (1.05-1.25)	1.15 (1.05-1.25)	1.07 (0.97-1.18)

*PYAR,* Person-years at-risk.

All models were fitted to patients with complete data for all included variables. Sets without at least 1 exposed and 1 unexposed individual were excluded. HRs were estimated from a Cox regression model with current age as the underlying timescale, stratified by matched set (sex, age, and general practice).

*Minimally adjusted*: N = 1,980,710 (1,920,172 unique people) in the depression cohort and N = 2,242,905 (2,171,784 unique people) in the anxiety cohort.

*Adjusted* for current calendar period and IMD at cohort entry (same participants as in minimally adjusted model).

*Additionally adjusted for potential mediators*: BMI (categorized as normal, 18.5-24.9 kg/m^2^; underweight, <18.5 kg/m^2^; overweight, 25.0-29.9 kg/m^2^; and obese, ≥30.0 kg/m^2^), smoking status, and alcohol and high-dose corticosteroid use (≥20 mg/d prednisolone equivalent dose), as time-updated variables. N = 1,371,005 individuals (1,322,284 unique people) in the depression cohort and N = 1,583,390 (1,583,390 unique people) in the anxiety cohort.

∗ *P* values were <.0001 for linearity in all models, and 0.3810, 0.3832, and 0.6983 for departure from linearity in minimally adjusted, confounder-adjusted-, and additionally adjusted for potential mediators models, respectively.

† *P* values were <.0001 for linearity in all models and <.0001 for departure from linearity in all models.

**Table E9:** Secondary analysis, adjusted models: association between atopic eczema and depression/anxiety, stratified on sex, current age (18-39, 40-59, 60+ y), and calendar period (1998-2001, 2002-2006, 2007-2011, 2012-2016)

Stratum/exposure	Depression	Anxiety	Adjusted HR∗ (99% CI)	No.	Events/PYAR	Adjusted HR∗ (99% CI)
	No.	Events/PYAR				
Sex†			*P* < .0001			*P* = .0003
Males						
No atopic eczema	785,368	3,8219/4,512,990	1.00 (reference)	841,681	25,390/4,806,123	1.00 (reference)
Atopic eczema	181,315	10,891/1,086,295	1.19 (1.16-1.23)	187,784	7,196/1,122,110	1.22 (1.17-1.27)
Females						
No atopic eczema	802,909	64,663/4,422,944	1.00 (reference)	977,115	56,747/5,381,375	1.000 (reference)
Atopic eczema	211,118	20,431/1,267,822	1.11 (1.08-1.13)	236,325	17,087/1,421,273	1.14 (1.11-1.17)
Current age†			*P* < .0001			*P* = .0052
18-39						
No atopic eczema	828,072	48,992/3,265,435	1.00 (reference)	938,376	39,939/3,696,223	1.00 (reference)
Atopic eczema	195,455	14,175/834,206	1.10 (1.06-1.13)	210,072	11,476/901,165	1.14 (1.10-1.18)
40-59						
No atopic eczema	485,291	26,208/2,553,247	1.00 (reference)	588,395	23,023/3,064,427	1.00 (reference)
Atopic eczema	123,489	8,624/675,274	1.22 (1.18-1.27)	138,864	7,031/756,141	1.21 (1.17-1.26)
≥60						
No atopic eczema	530,344	27,682/3,117,252	1.00 (reference)	595,827	19,175/3,426,849	1.00 (reference)
Atopic eczema	139,919	8,523/844,638	1.12 (1.08-1.16)	149,256	5,776/886,077	1.15 (1.10-1.21)
Calendar period†			*P* = .3229			*P* = .2871
1998-2001						
No atopic eczema	476,794	17,175/1,143,231	1.00 (reference)	533,384	12,824/1,274,519	1.00 (reference)
Atopic eczema	113,944	4,888/279,596	1.14 (1.09-1.19)	121,449	3,499/297,984	1.15 (1.09-1.21)
2002-2006						
No atopic eczema	762,671	28,735/2,341,702	1.00 (reference)	863,959	22,307/2,652,645	1.00 (reference)
Atopic eczema	187,774	8,525/595,943	1.15 (1.11-1.19)	201,836	6,298/642,215	1.15 (1.10-1.19)
2007-2011						
No atopic eczema	1,000,682	32,533/3,137,512	1.00 (reference)	1,148,842	24,850/3,589,229	1.00 (reference)
Atopic eczema	256,158	9,916/836,225	1.12 (1.08-1.16)	277,719	7,441/905,501	1.18 (1.13-1.22)
2012-2016						
No atopic eczema	923,647	24,439/2,313,488	1.00 (reference)	1,068,458	22,156/2,671,107	1.00 (reference)
Atopic eczema	248,593	7,993/642,355	1.15 (1.12-1.20)	270,531	7,045/697,684	1.19 (1.14-1.24)

*PYAR*, Person-years at-risk.

∗ All models were fitted to individuals with complete data for all included variables. Sets without at least 1 exposed and 1 unexposed individual were excluded. HRs were estimated from a Cox regression model with current age as the underlying timescale, stratified by matched set (sex, age, and general practice), and adjusted for current calendar period (years: 1998-2001, 2002-06, 2007-11, and 2012 16) and quintiles of IMD at cohort entry.

† *P* values for interaction and for stratum-specific effect were derived through likelihood-ratio tests.

## Online Repository Methods

### Codes and treatments used in algorithm definition of atopic eczema

Using a validated algorithm,^E1^ atopic eczema diagnosis was determined by a combination of any recorded atopic eczema diagnostic code in primary care (CPRD, using Read codes) or inpatient hospital admission data (HES, using *International Classification of Diseases, Tenth Revision* codes recorded in any diagnostic position of any episode of care), and 2 atopic eczema therapies (recorded on separate days in primary care or hospital records).

To avoid immortal-time bias, the date of diagnosis was set as the date of the latest recorded component (ie, atopic eczema diagnostic code or second record for atopic eczema therapy) in the algorithm.

By default, all individuals with atopic eczema were classified as having mild disease. They could be recategorized as having moderate atopic eczema from the earliest of (1) second potent topical steroid treatment within a year or (2) first calcineurin inhibitor treatment. Mild or moderate cases could be recategorized as severe, from the earliest of (1) first systemic treatment for atopic eczema excluding oral glucocorticoids (ie, a record of a prescription for cyclosporine, azathioprine, mycophenolate, or methotrexate), or (2) first phototherapy code, or (3) first referral to secondary care for atopic eczema.

Using electronic health records allowed us to conduct a population-based analysis powered to explore the association between atopic eczema, and depression and anxiety. However, the analysis was limited to data routinely captured in primary care, which does not include objective assessment of patients' eczema disease severity using validated scoring systems (eg, Eczema Area and Severity Index and SCORing Atopic Dermatitis).^E2^ To date, there are no validated measures for ascertaining the severity of atopic eczema using routinely collected data.^E3^ However, the approach we used to overcome this limitation (ie, use of prescribed therapies) is commonly used in the dermato-epidemiology literature.E4, E5, E6, E7, E8, E9

In the United Kingdom, for example, systemic treatments may be initiated only by dermatologists,^E10^ making this proxy measure a highly specific one, because it implies assessment by a trained specialist. Although this method may misclassify individuals with severe eczema who declined therapy,^E11^ findings from secondary care registries suggest that 60% to 80% of specialist-diagnosed severe eczema cases did have a history of systemic treatment.E12, E13 Findings from recent publications also support the validity of this approach: A positive predictive value of 93.6% was demonstrated for the diagnosis of severe atopic eczema using Israeli health records,^E4^ and a Danish validation study assessing a similar approach among patients with psoriasis suggested 91% sensitivity and 83% specificity for detection of severe disease.^E14^

Relying on prescribed therapies as a proxy measure for disease severity may not be fully calibrated with other established scores, making severity-stratum–specific estimates harder to interpret. However, a prescribed treatment criterion is likely to perform well in separating those with a more severe manifestation (ie, good discrimination). Because of the prospective nature of data collection in our study, it seems unlikely that any misclassification of eczema severity would be differentially associated with the future outcome, nor does it seem to be of a magnitude large enough to substantially bias the effect estimate. In such circumstances, an estimate of trend would be biased downwards,^E15^ which would only strengthen the validity of a demonstrated biological gradient.

Our choice of treatments used to define moderate and severe atopic eczema is broadly consistent with previous similar attempts,E4, E5, E6, E7, E8, E9 but also reflects some international variability in prescription practices. For example, although topical calcineurin inhibitors are indeed recommended for use in mild disease by recent European guidelines,^E16^ they are not used for this indication in the United Kingdom (where our study is set). The European Medicines Agency has issued a recommendation for cautious use of topical tacrolimus and pimecrolimus due to potential risks of skin cancer and lymphoma.^E17^ In the United Kingdom, these preparations are not considered as first-line options for mild atopic eczema, and they can be initiated only by specialistsE10, E18; consequently, we used a prescription for a calcineurin inhibitor as a marker of moderate eczema.

Read codes and *International Classification of Diseases* codes defining atopic eczema and atopic eczema treatments can be downloaded.^E19^

#### Drugs used for treating eczema

• emollients
• topical glucocorticoids
• topical tacrolimus and pimecrolimus
• oral glucocorticoids
• azathioprine
• ciclosporin
• methotrexate
• mycophenolate mofetil

### Code lists for the outcomes (depression and anxiety)

#### Development of code lists

The monitoring and management of depression is financially incentivized in UK primary care through the Quality and Outcomes Framework,^E20^ which uses a list of Read codes to define new occurrences of depression.^E21^ This resulted in some standardization of codes used in general practice, but was also highlighted as a contributing reason for a growing trend among general practitioners to use symptom codes for depression (eg, “depressive symptoms” and “Low mood”) rather than definitive diagnostic codes (eg, “major depression”).E22, E23 Symptom codes are also increasingly used by general practitioners in the United Kingdom for patients with anxiety, despite the fact that anxiety diagnosis and treatment are not included in the Quality and Outcomes Framework. This trend possibly reflects diagnostic uncertainty and a reluctance to stigmatize patients.E24, E25

We compiled preliminary lists of potential codes using keywords from the Medical Subjects Headings list and clinical knowledge, as previously suggested.E26, E27 This initial list of codes was compared with and augmented by a code list from the Clinical research using Linked Bespoke studies and Electronic health Records website (a platform that aims to offer researchers predefined code lists and algorithms to identify clinical phenotypes using electronic health record data)^E28^ and previously published lists used to define depressionE21, E29, E30, E31, E32 and anxiety.E29, E31, E32, E33 Subsequently, we categorized the relevant codes into several subcategories, on the basis of clinical knowledge, literature review, and the expert opinion of several coauthors experienced in general practice (Y.S.), UK clinical practice and EHR research (S.M.L., J.F.H., and K.E.M.), and psychiatry and psychiatric epidemiology using electronic health record data (J.F.H.). The list was finalized through a discussion and consensus process.

Codes for depression were categorized as follows:

1. “Core” codes: Compatible with “classic unipolar” depression diagnoses, of various severities.^E34^ Broadly following the Quality and Outcomes Framework–eligible diagnoses,^E21^ and in line with previous publications.E29, E30, E31, E32
2. Symptom codes.
3. Nondefinitive codes: Including broader definitions of depression, depression due to transient causes, and depression-related administrative codes.
4. History codes: Asserting a history of depression

We used a similar framework to define incident anxiety cases. Codes compatible with generalized anxiety disorder and panic disorder were considered as “core” diagnoses. Codes for specific phobias were included in the nondefinite category.E24, E29, E32, E33

We used codes classified as core codes to define the primary outcomes in the main analyses and explored the possible introduction of bias through sensitivity analyses (Table E2).

Individuals were excluded from the depression or anxiety cohort after matching if they had a relevant “core,” “symptom,” or “nondefinitive” code recorded at any time before cohort entry (ie, those with previous depression were excluded from the depression cohort, and those with previous anxiety were excluded from the anxiety cohort). Individuals with a code for a clinical term including a “history of” anxiety or depression recorded at any time were also excluded from the relevant cohort, because the timing of their diagnosis could not be ascertained.

The recording of our depression/anxiety outcomes is likely to be biased among those with codes compatible with alternative etiologies for the outcome (ie, organic depression or dementia for the depression cohort, obsessive-compulsive disorder or posttraumatic stress disorder for the anxiety cohort, and schizophrenia or bipolar disease for both cohorts). We, therefore, excluded people with these conditions if diagnosed before the index date, and censored participants upon a subsequent diagnosis (such alternative diagnoses were, by an order of magnitude, less frequent than depression/anxiety). If excluded individuals were in the atopic eczema–exposed group at baseline, their respective matched unexposed individuals were also excluded.

Read codes and *International Classification of Diseases* codes can be downloaded.^E19^

Read codes used to define bipolar disease and schizophrenia were taken from the Clinical research using Linked Bespoke studies and Electronic health Records website.^E28^

### Directed acyclic graph

A review of the literature revealed several conditions associated with atopic eczema: age,E1, E35, E36, E37, E38, E39 female sex,E36, E37, E38, E39, E40, E41 socioeconomic status,E35, E36 ethnicity, health care interactions/health anxiety,E35, E41, E42, E43 obesity,^E44^ smoking status,^E36^ alcohol use,E36, E38, E45 diabetes,E8, E36, E46 malignancies,E36, E46 and chronic conditions (including asthma, cardiovascular diseases, attention deficit/hyperactivity disorder, rheumatoid arthritis, renal diseases, and inflammatory bowel disorders).E7, E8, E36, E46, E47 To guide covariate selection *a priori*, and to avoid collider bias, we constructed a directed acyclic graph (Figure E2).^E48^

*Chronic diseases* could play a mediating role in the association between atopic eczema and depression/anxiety, but could also be more likely to be diagnosed among those with depression/anxiety, and therefore introduce collider bias. Chronic comorbidities were, therefore, not included in our analyses.

A potential confounder (1) must be a risk factor for the outcome (ie, a cause, or surrogate for a cause, of depression/anxiety); (2) must be associated with the exposure (ie, atopic eczema); and (3) cannot be an intermediate step on the causal path between exposure and outcome (ie, it must not mediate the association between atopic eczema and depression/anxiety) nor be a consequence of the outcome (depression/anxiety).^E49^ Although some chronic conditions may be associated with atopic eczema,^E50^ as well as with depression/anxiety,^E51^ in the context of this study, we did not consider that these chronic conditions fit the definition for confounding because the potential confounder (chronic comorbidity) could be considered to either be a consequence of the outcome (anxiety/depression) or to mediate the relationship between exposure and outcome. Even though our study specifically excluded preexisting psychiatric diagnoses, there is a possibility of undiagnosed mental illness, so chronic conditions could still be potential consequences of anxiety or depression because (1) most primary care patients with depression initially present with somatic symptoms,^E52^ and increased general practitioner attendance is associated with earlier diagnosis of chronic conditions,E53, E54 and (2) depression/anxiety (ie, the outcomes) are well-established independent risk factors for chronic medical conditions.E55, E56, E57, E58 Therefore, we did not adjust for chronic comorbidities as confounders, because adjusting for factors that may be influenced by both the exposure and the outcome would introduce collider bias.E49, E59

Alternatively, we could regard chronic comorbidities as mediators of the relationship between atopic eczema (exposure) and anxiety/depression (outcomes). By adjusting for lifestyle factors (ie, BMI, smoking, and harmful alcohol use) as potential mediators, we were able to capture, at least some of, the effect of atopic eczema on chronic comorbidities (ie, considering lifestyle factors as being on the causal pathway between atopic eczema and chronic comorbidities such as cardiovascular disease). Of note here, additional adjustment for potential mediators (Table II) only slightly attenuated the effect of atopic eczema on anxiety/depression, suggesting that further adjustment for chronic comorbidities may have limited impact. So, although atopic eczema may predict conditions such as cardiovascular disease and various malignancies,E7, E50 exploring the potential mediating role of chronic comorbidity was beyond the scope of our analysis.

### Definitions for included covariates

The date of birth was set as July 1 for all participants, because CPRD supplies year of birth only (to ensure patient anonymity). Calendar period was categorized as 1998 to 2001, 2002 to 2006, 2007 to 2011, and 2012 to 2016, to account for changes in clinical, diagnostic, and administrative practices over the study period that may have influenced the measurement of exposure, outcomes, and other covariates. Socioeconomic deprivation was assigned through categorizing each participant's 2007 Index of Multiple Deprivation (IMD) score categorized in quintiles. The IMD is an index of deprivation updated by the Department for Communities and Local Government every few years, combining information from 7 domains: income, employment, health and disability, education, barriers to housing and services, living environment, and crime.^E60^ IMD data are available for the years 2004, 2007, 2010, and 2015. We chose 2007 as the midpoint of the study (January 1998 to March 2016). IMD was assigned on the basis of individual postcode of residence, or on 2010 practice-location if other data were unavailable.

BMI was categorized as underweight (<18.5 kg/m^2^), normal (20-24 kg/m^2^), overweight (25-29 kg/m^2^), and obese (>30 kg/m^2^), in line with the World Health Organization categorization.^E61^ Harmful alcohol use was based on relevant morbidity coding or prescriptions for drugs used to maintain alcohol abstinence, with status changing at first primary care record suggesting harmful alcohol use. High-dose oral glucocorticoid use was defined as greater than or equal to 20 mg/d prednisolone equivalent dose, for the duration of the patients' prescription and 3 months after the end of the prescription (glucocorticoids included deflazacort, dexamethasone, prednisone, prednisolone). Smoking status and BMI were defined on the basis of status recorded closest to the date an individual entered the cohort. Missing doses of glucocorticoids were completed by using data from the entire data set. (See "Algorithms to identify BMI and steroid use data" for a full description of the data completion algorithms.)

For a sensitivity analysis, ethnicity was included as covariate, based on a previously validated algorithm.^E62^ Ethnicity was assigned to 5 categories: white, South Asian, black, other, or mixed. First, the most common ethnicity in CPRD was used, then the latest ethnicity in CPRD was used where several ethnicities were recorded equally, and finally HES ethnicity was used where CPRD ethnicity was missing. Because the quality of the recording for ethnicity was acceptable only from 2006 onwards,^E62^ we restricted this analysis to those registering with the CPRD general practice after January 1, 2006.

Read codes and *International Classification of Diseases* codes used to identify covariates can be downloaded.^E19^

### Algorithms to identify BMI and steroid use data

#### Body mass index

Read codes for BMI category were not used (because they are rarely recorded). Instead BMI was calculated using height and weight measures recorded closest to the cohort entry date (1 year before to 1 month after the cohort entry date; if not available, then the nearest in the year following cohort entry was taken; if not available, then the nearest value in the year before cohort entry was taken; if not available, then nearest weight value recorded after the first year was used). We regarded improbable values of BMI (BMI < 10 kg/m^2^ or BMI > 60 kg/m^2^) as errors, and therefore considered these data as missing. Smoking status was categorized as current/ex or none on the basis of record closest to cohort entry date.

#### Glucocorticoid dose

The daily dose of oral glucocorticoid was calculated as the number of pills prescribed numeric daily dose (NDD) × dose per tablet. Where NDD was missing, a “hot-deck” style imputation method was adopted, which replaced missing data with comparable data from the same set. An extra binary variable for quantity of tablets per prescription was created, categorizing quantity about the median number into low and high. If a patient had any other record with the same quantity and dose per tablet, the median NDD among those records was used where NDD was missing. If a patient had no recorded NDD but had any other record of the same dose per tablet and quantity as a binary variable, the median NDD among those records was used. If a patient did not have a recorded NDD or quantity, but had records for the same dose per tablet, then the median NDD among those records was used. If there was no record of NDD, dose per tablet or quantity, but there were other patients in the data set in the same 5-year age band, of the same sex, with the same dose per tablet and quantity, the median NDD for those records was used. Finally, if none of the above were possible, patients in the data set in the same 5-year age band, of the same sex, with the same dose per tablet and quantity as a binary variable, the median NDD among these records was used.
